# Growth Dynamics of the Threatened Caribbean Staghorn Coral *Acropora cervicornis*: Influence of Host Genotype, Symbiont Identity, Colony Size, and Environmental Setting

**DOI:** 10.1371/journal.pone.0107253

**Published:** 2014-09-30

**Authors:** Diego Lirman, Stephanie Schopmeyer, Victor Galvan, Crawford Drury, Andrew C. Baker, Iliana B. Baums

**Affiliations:** 1 Rosenstiel School of Marine and Atmospheric Science, University of Miami, Miami, Florida, United States of America; 2 Punta Cana Ecological Foundation, Punta Cana, Dominican Republic; 3 Pennsylvania State University, State College, Pennsylvania, United States of America; National University of Singapore, United States of America

## Abstract

**Background:**

The drastic decline in the abundance of Caribbean acroporid corals (*Acropora cervicornis*, *A. palmata*) has prompted the listing of this genus as threatened as well as the development of a regional propagation and restoration program. Using *in situ* underwater nurseries, we documented the influence of coral genotype and symbiont identity, colony size, and propagation method on the growth and branching patterns of staghorn corals in Florida and the Dominican Republic.

**Methodology/Principal Findings:**

Individual tracking of> 1700 nursery-grown staghorn fragments and colonies from 37 distinct genotypes (identified using microsatellites) in Florida and the Dominican Republic revealed a significant positive relationship between size and growth, but a decreasing rate of productivity with increasing size. Pruning vigor (enhanced growth after fragmentation) was documented even in colonies that lost 95% of their coral tissue/skeleton, indicating that high productivity can be maintained within nurseries by sequentially fragmenting corals. A significant effect of coral genotype was documented for corals grown in a common-garden setting, with fast-growing genotypes growing up to an order of magnitude faster than slow-growing genotypes. Algal-symbiont identity established using qPCR techniques showed that clade A (likely *Symbiodinium* A3) was the dominant symbiont type for all coral genotypes, except for one coral genotype in the DR and two in Florida that were dominated by clade C, with A- and C-dominated genotypes having similar growth rates.

**Conclusion/Significance:**

The threatened Caribbean staghorn coral is capable of extremely fast growth, with annual productivity rates exceeding 5 cm of new coral produced for every cm of existing coral. This species benefits from high fragment survivorship coupled by the pruning vigor experienced by the parent colonies after fragmentation. These life-history characteristics make *A*. *cervicornis* a successful candidate nursery species and provide optimism for the potential role that active propagation can play in the recovery of this keystone species.

## Introduction

One of the Caribbean's predominant reef-building coral genera, *Acropora*, has suffered significant population declines caused by multiple natural and anthropogenic stressors over the past three decades [1], [2], [3]. The loss of *Acropora* has resulted in decreases in reef function and structure as acroporid corals are critically important for reef growth, island formation, fisheries habitat, coastal buffering, and biodiversity [4]. The significant decline in abundance of this keystone taxon has prompted a number of conservation measures aimed at protecting remaining populations and accelerating its recovery trajectory. These efforts include: 1) the recent listing of both species of Caribbean *Acropora* as threatened under the US Endangered Species Act [5], and 2) the development of regional propagation and restoration programs [6].

The active restoration projects developed for the Caribbean staghorn coral (*A. cervicornis*) have provided an opportunity to explore the early growth of staghorn fragments and colonies at an unprecedented level of detail. In this study, we report on the growth dynamics of >1700 staghorn individuals representing 37 distinct genotypes from Florida and the Dominican Republic that were followed from the time of collection/fragment creation to the development of complex branching morphology. While the growth patterns of *Acropora palmata* and *A. cervicornis* were documented in the past, growth was only reported based on linear extension of a subset of branches within colonies [7], [8], [9], and no information on the role of coral genotype was previously examined, except for the recent study by Griffin et al. [10] that reported on the growth of six staghorn genotypes in Puerto Rico.

Coral growth is the ultimate indicator of coral health, integrating the influence of internal factors such as coral genotype and symbiont identity, physiology, and external environmental factors. However, growth is often difficult to estimate for corals with complex or irregular morphologies. Colony sizes, in such cases, are often approximated to regular geometric shapes [11] that are then used to estimate surface area and colony volume, metrics commonly related to habitat value [12]. While these approximations are adequate to document higher-level metrics like coral cover or colony size-frequencies, they are not adequate, due to measurement error, to quantify colony growth accurately, especially for branching species like staghorn corals.

Coral growth was measured here as Total Linear Extension (TLE), a painstaking procedure that quantifies all of the tissue/skeleton on each individual. Using this unique dataset, we explore the role of fragment size, coral genotype, and symbiont identity on growth. The data presented here not only help elucidate the factors that influence growth at early stages of the asexual propagation process but also provide important guidelines for the propagation of *Acropora* within nursery settings to maximize the productivity of this important and threatened Caribbean taxon.

## Materials and Methods

### Coral Growth and Productivity

Total Linear extension (TLE, [13]) was estimated for each individual using a flexible ruler at the time of deployment and again after 12 months. Measurements included all branches and were calculated to the closest cm (Fig. S1). In addition to TLE, the number of terminal branches (> 1 cm in length) was assessed. When the measurements for a specific batch of fragments deviated > 1 week from the scheduled interval due to unfavorable field conditions, TLE values were extrapolated linearly to calculate 12-month values. Change in TLE over time (growth) was estimated as the total amount of tissue/skeleton produced between measurement intervals. In addition to growth, annual productivity was estimated as the amount of coral produced relative to the tissue/skeleton present at the start of the study (annual productivity  =  growth/TLE at start of study). Only fragments that were alive for the 12-month period and did not undergo partial tissue mortality or fragmentation were included in the calculations. This 1-year period was selected to have a uniform growth interval to compare among the different nursery programs that conducted fragmentation and transplantation at different times of the year or in different years, and to be able to track fragments from the time they were created as “fingerlings” with a simple morphology of 1–2 branches, to the time when complex branching morphology developed, marking the transition from fragments to colonies [14], [15]. The measurement of TLE becomes increasingly difficult as colonies develop multiple branches [11] and fragmentation becomes more frequent due to physical impacts of fishing gear and storms, affecting growth measurements.

Each coral genotype was represented by multiple fragments that were labeled with either a colored cable tie or with numeric tags and measured individually. Growth and productivity data were compared: 1) among coral genotypes; 2) between fragments growing in an underwater nursery and natural reefs; and 3) among fragments growing on different nursery propagation platforms (i.e., frames, floating ropes). The growth and productivity data were log-transformed (log_10_ (x+1)) due to lack of normality of some of the fragment groups. The assumptions of normality and homoscedasticity were tested for each comparison and, when the transformed data were either not normal or heteroscedastic, non-parametric tests were used instead of parametric tests.

Previous work by Lirman et al. [16] showed that the removal of small fragments (3–5 cm) appears to stimulate the growth of donor branches on parent colonies (after a brief initial hiatus in growth as the skeletal lesions heal and new apical polyps develop). However, this conclusion was based on the removal of <10% of the parent tissue. In the present study, we evaluated the impacts of high levels of fragmentation (up to 95% of the tissue removed) to determine whether the vigor associated with minor fragmentation is still observed when larger portions of colonies are removed. This was achieved by comparing the growth of colonies before and after pruning.

### Study Sites

**Figure 1:**
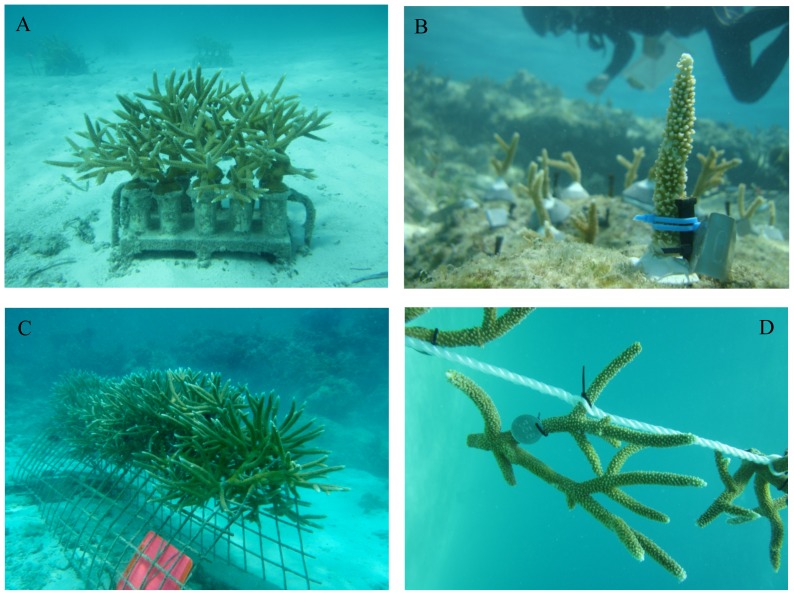
Staghorn fragments propagated within *in situ* nurseries. A) Cinder-block platform used to propagate corals in Florida, B) coral outplant attached to a reef using a masonry nail, epoxy, and plastic ties, C) A-frame used to propagate corals in the Dominican Republic, D) rope nursery.

Sites in Florida included two underwater nurseries (25.488° N, 80.109° W; 25.362° N, 80.166° W) as well as 5 reefs where nursery-grown corals were outplanted and monitored (Site 1: 25.465° N, 80.149° W; Site 2: 25.462° N, 80.142° W; Site 3: 25.361° N, 80.177° W; Site 4: 25.357° N, 80.169° W; Site 5: 25.365° N, 80.171° W). The nurseries were established on sandy substrate at a depth of 5–6 m. Fragments in the two Florida nurseries were attached to cinder blocks containing PVC pedestals using epoxy (Fig. 1A). The blocks (20×40×10 cm) contained 10 fragments of a single genotype each and were spaced at a minimum distance of 1 m between blocks. Nurseries in Florida contained 40–50 blocks and 400–500 fragments, occupying an area on the bottom of roughly 20 m×20 m. Fragments within blocks were separated by 8–10 cm at the start of the study. Nursery-grown fragments were outplanted to 5 Florida reefs (4–5 m) using masonry nails pounded onto the reef substrate and attached to the nails using plastic cable ties (Fig. 1B). Underwater epoxy was used to further cement the nails and the bases of fragments onto the reef. The reef sites were separated by a minimum distance of 700 m. At each site, replicates (10–15 from each genotype) from multiple genotypes collected from the nurseries were deployed in a regular grid containing 50–100 corals at a spacing of 50–100 cm between fragments, with each grid covering an area of 50–100 m^2^. Replicates of each coral genotype were haphazardly located within each plot.

The staghorn nursery and 9 outplant sites at the Dominican Republic (DR) are located at Punta Cana (68.347° W, 18.539° N). The nursery is located on sandy substrate (4–5 m of depth) and covers an area of approximately 30 m×30 m. Fragments are grown on metal frames secured to the bottom or on floating ropes (Fig. 1C, D). Multiple genotypes and numerous replicate fragments of each genotype are grown on each frame and rope. The Punta Cana nursery contained 14 frames and 3 ropes, holding> 1300 fragments. Initially (when fragments are small), frames and ropes can hold up to 100 fragments. Initial spacing of fragments within frames and ropes is approximately 20 cm. Corals from the nursery were outplanted to 9 sites located along a continuous reef in the Punta Cana region (depth  =  4–6 m). Sites were separated by a minimum of 200 m. At each site, corals were attached to the bottom using similar methods to those used in Florida [13]. Each site contained a single plot of 50–80 fragments but only a subset of those corals was monitored for growth (15–20 fragments per site). Corals were deployed in a regular grid at a spacing of 50–100 cm between fragments, with each grid covering an area of 50–100 m^2^. Between 15–20 replicates of each coral genotype were deployed at each site and fragments were haphazardly located within each plot.

Activities in Florida were conducted under permit BISC-2010-SCI-0008 provided by Biscayne National Park and Special Activity License SAL-10-1086-SCRP provided by the Florida Fish and Wildlife Conservation Commission. All activities conducted with *Acropora*, a threatened species listed under the US Endangered Species Act (ESA), were approved by a Section 7 Consultation Biological Opinion issued by The National Oceanic and Atmospheric Administration (NOAA) and the National Marine Fisheries Service (NMFS). Activities in the Dominican Republic were conducted under a permit provided by Ministerio de Medio Ambiente y Recursos Naturales (Ministry of the Environment and Natural Resources).

### Coral Genotype Identity

For genetic analyses, a small section (1–2 cm) from a branch tip was removed from each target colony and preserved in 95% ethanol. DNA was extracted from each sample using the Qiagen DNEasy 96 blood and tissue kit (Qiagen, CA). Microsatellite markers were those used by Baums et al. [17], [18] developed originally for *Acropora palmata.* Here, we used markers 166, 181, 182 and 207. Marker 192 does not amplify in *A. cervicornis*. PCR conditions were as in Baums et al. [19]. Thermal cycling was performed in an MJ Research PT200 or an Eppendorf Mastercycler Gradient cycler. Alleles were fluorescently labeled and then visualized and sized with internal standards on a PRISM 3100 Genetic Analyzer (Applied Biosystems). Electropherograms were visualized and allele sizes were called using GENEMAPPER 4.0 (Applied Biosystems). The microsatellite markers are highly heterozygous (mean observed heterozygosity  =  0.77), thus there is a low probability of identifying two colonies or cells as clonemates when in fact they are distinct genets (this is called the Probability of Identity (PI) and equals 10^−7^ for *A. cervicornis.* These values were estimated for a dataset containing 278 samples from throughout the Caribbean [19]. We identified clonemates in the Florida dataset by comparing all new multilocus genotypes against the existing *A. cervicornis* genotypes (generated in the same lab under identical conditions, Baums et al., 2009) and against each other. Those colonies that shared identical alleles at all loci were deemed to be clonemates. Matching calculations were performed usingGenAlEx vers 6.4 [20].

### Algal Symbiont Identity

Algal symbiont communities in genetic samples were characterized by extracting DNA following established protocols [21] and using an actin-based quantitative PCR (qPCR) assay to detect *Symbiodinium* in clades A, B, C and D. Primers and probes that targeted each clade, as well as the coral host, were modified from Mieog et al. [22] following the approach of Cunning and Baker [23]. Each sample was run in duplicate, with positive and no-template controls. This assay allowed us to identify the dominant *Symbiodinium* in the study corals at the clade level, as well as detect any symbionts present at low abundance (<10%).

## Results

### Effects of Colony Size

**Figure 2:**
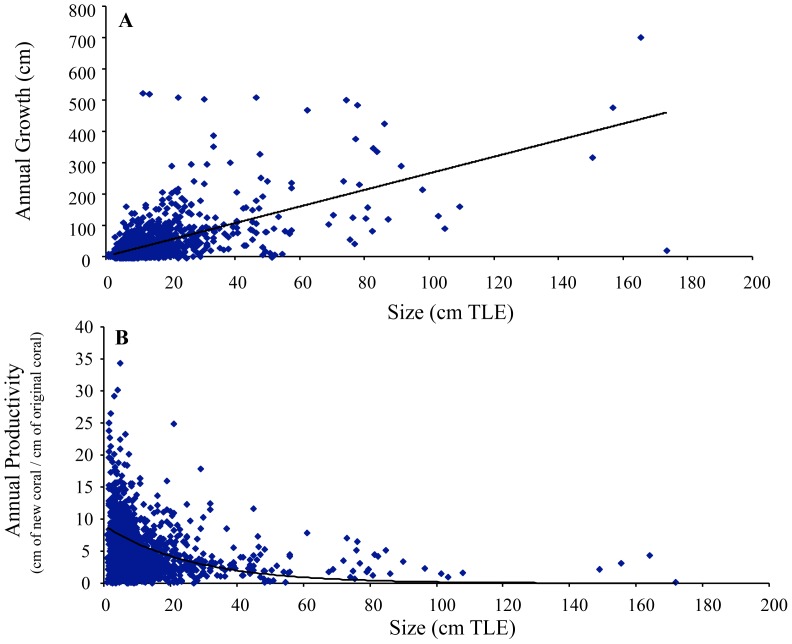
Growth and productivity of staghorn fragments. Annual growth (A) and annual productivity (B) of staghorn fragments.

A total of 1715 fragments were measured in this study. Fragments grown and outplanted in the DR were generally larger than those used in Florida so, for comparison, growth and productivity data were calculated for four size classes: tips (<5 cm TLE), small (5 – 15 cm TLE), medium (>15 – 30 cm TLE), and large (> 30 cm TLE) (Table 1). While growth increased with increasing fragment size in both nursery and reef settings, productivity decreased with increasing fragment size in both Florida and the DR (Table 1). Growth was significantly and positively related to TLE recorded at the beginning of the growth interval across a wide range of initial sizes (1–175 cm TLE) (linear regression, p<0.05, R^2^ = 0.37, n = 1715) (Fig. 2A). Moreover, a significant positive linear relationship was documented between TLE and number of branches after 12 months of growth (linear regression, p<0.05, R^2^ = 0.16). Productivity declined exponentially with increasing initial colony size, with maximum annual productivity recorded for fragments <15 cm in TLE (non-linear regression, p<0.05, R^2^ = 0.34) (Fig. 2B).

**Table 1:** Annual growth and productivity (± S.D.) of staghorn fragments in Florida and the DR.

Location	Type	Size Category	N fragments	Growth (cm per year)	Annual Productivity (growth/cm of initial coral)
DR	Nursery	Tips	*	*	*
DR	Nursery	S	130	59.3 (68.9)	5.6 (6.4)
DR	Nursery	M	156	97.2 (80.3)	4.5 (3.6)
DR	Nursery	L	94	173.1 (136.3)	3.3 (2.4)
DR	Reef	Tips	5	20.1 (15.9)	4.2 (3.1)
DR	Reef	S	72	29.7 (26.9)	3.0 (2.5)
DR	Reef	M	46	49.6 (43.2)	2.4 (1.9)
DR	Reef	L	*	*	*
Florida	Nursery	Tips	446	23.7 (16.1)	7.0 (5.1)
Florida	Nursery	S	140	48.5 (32.2)	6.0 (3.6)
Florida	Nursery	M	10	89.3 (29.5)	4.6 (1.4)
Florida	Nursery	L	*	*	*
Florida	Reef	Tips	75	28.1 (31.6)	5.9 (6.5)
Florida	Reef	S	349	36.5 (28.1)	4.2 (3.3)
Florida	Reef	M	25	71.1 (45.9)	4.0 (2.7)
Florida	Reef	L	*	*	*

*  =  <5 fragments of that size class were used.

Tips  =  <5 cm TLE, S  =  5–15 cm TLE, M  = >15 – 30 cm TLE, L  = > 30 cm TLE.

### Effects of Coral Genotype and Algal Symbiont Identity

**Figure 3:**
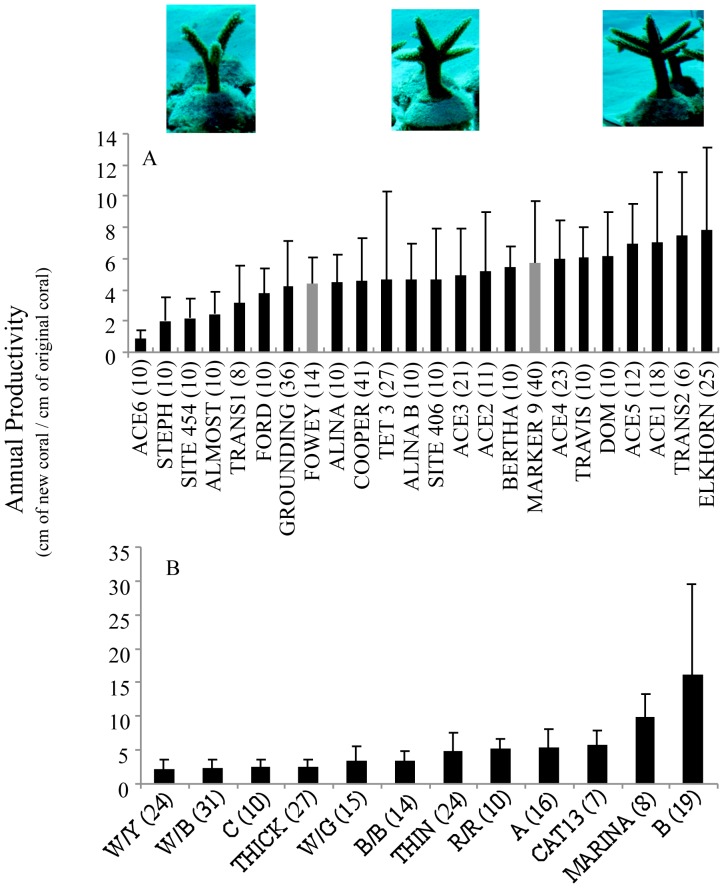
Productivity of staghorn fragments by coral genotype. Annual productivity of staghorn genotypes from Florida (A) and the Dominican Republic (B). Numbers in parentheses are sample sizes for each genotype. Grey bars in (A) are the two genotypes dominated by clade C algal symbionts. All other genotypes, including those in the DR were dominated by clade A. The coral genotype from the DR dominated by clade C was represented by only 3 surviving fragments and thus not included in the analyses. The images are representative of slow- (left), medium- (center), and fast-growing (right) genotypes after 1 year in the nursery. Pair-wise comparisons revealed that only ELKHORN and ACE 6/STEPH had significantly different productivity values in Florida, while only B and W/B/W/Y had significantly different productivity values in the DR (Tukey-Kramer test, p<0.05).

A total of 37 distinct coral genotypes were identified, 24 from Florida and 13 from the DR. The growth of individual coral genotypes was documented in nurseries in Florida (6–41 fragments per genotype) and the DR (7–31 fragments per genotype) over 1 year. Annual productivity values ranged from 0.9–9.8 cm of new coral produced by every cm of coral present at the start of the study for the 24 genotypes examined in Florida (Fig. 3A). In the DR, the range of annual productivity values was 2.2–16.2 cm for the genotypes examined (Fig. 3B). Significant differences in productivity were detected among genotypes in Florida and in the DR (Mann Whitney U test, p<0.05), but pair-wise comparisons revealed that only the fastest (ELKHORN in Florida, and B in the DR) and slowest genotypes (ACE 6 and STEPH in Florida, and W/B and W/Y in the DR) had significantly different productivity values (Tukey-Kramer test, p<0.05).

In both Florida and the DR, qPCR analysis revealed that *Symbiodinium* in clade A (likely *Symbiodinium* A3) was the dominant symbiont for all coral genotypes, except for one coral genotype in the DR (out of 13) and two coral genotypes in Florida (out of 24) that were dominated by clade C (Fig. 3A). In Florida, there were no significant differences in growth or productivity between corals dominated by *Symbiodinium* in clade C (2 coral genotypes) and corals dominated by clade A symbionts (22 coral genotypes) (t test, p>0.05 for all metrics). The coral genotype from the DR dominated by clade C was represented by only 3 surviving fragments and thus not included in the analyses. In the DR, qPCR analysis found that *Symbiodinium* in clades B and C were also commonly found at low abundance in colonies dominated by clade A, but clade D was never detected in any of these samples. In Florida, 8 out of 17 genotypes that were dominated by clade A also contained clade C at low abundance, with one genotype also containing clade D (likely D1a). Of the 2 genotypes that were dominated by clade C, one also contained low-abundance A and D, while the other contained low-abundance A and B.

### Effects of Propagation Platform

A variety of propagation platforms are used in nursery programs to grow staghorn coral [13]. The platforms are classified into two types, fixed to the bottom (Fig. 1A, C) or floating (Fig. 1D). Here, we evaluate differences in growth metrics for small and medium fragments (the size categories represented in both ropes and frames) grown on wire frames (n = 259) and floating ropes (n = 27) in the DR. The same genotypes were grown on both platforms. Corals grown suspended on ropes had significantly higher annual productivity (6.6 cm (S.D. = 3.7) and number of branches per colony (5.7 (S.D. = 4.5)) than corals grown on frames fixed to the bottom (4.8 cm (S.D. = 5.1); 4.4 branches (S.D. = 4.7)) (t test, p<0.05 for each metric).

### Effects of Habitat

Nursery-grown corals were outplanted to natural reefs in Florida, providing the opportunity to compare growth metrics between the nursery (Fig. 1A) and reefs (Fig. 1B). For fragments within the “small” category (the size category well represented in both the nursery and reef) (n = 140 nursery, n = 349 reef outplants),corals grown on blocks within the nursery had significantly higher annual productivity (6.0 cm (S.D. = 3.6)) than corals transplanted to reefs (4.2 cm (S.D. = 3.3)) (t test, p<0.05).

### Effects of Pruning

**Figure 4:**
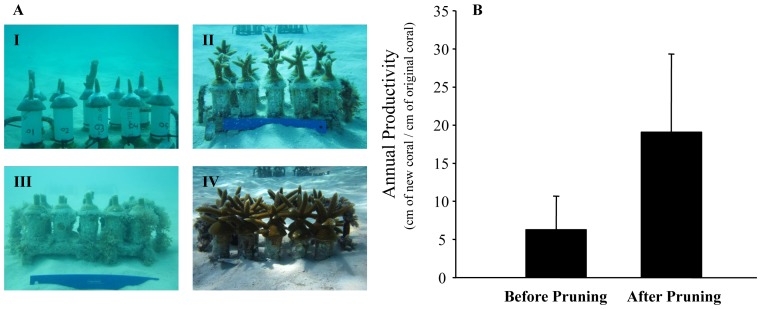
Effects of pruning on nursery corals. A) I: Fragments after initial collection, II: fragments 8 months after initial collection, III: fragments immediately after pruning (12 months after initial collection), IV: fragments 8 months after pruning. B) Annual productivity of fragments before pruning (after initial collection) and after pruning. Significant difference in productivity were found between groups (t test, p<0.05).

The growth of fragments from 19 coral genotypes (n  =  5–10 fragments per genotype) within the Florida nurseries was assessed for 1 year after collection (n  =  402 fragments). After the first year of growth, the resulting colonies were pruned to produce a second generation of fragments that were used for outplanting activities. At this time, 40–95% of each colony was trimmed. The growth of the pruned colonies (n  =  164 fragments) was then followed for an additional year to document the impacts of pruning on growth metrics (Fig. 4A). The average coral size at collection was 4.8 cm (S.D. = 2.0) compared to a lower average size immediately after pruning (3.2 cm (S.D. = 1.6)). The average number of branches per colony at collection and immediately after pruning was the same, 1.3 branches per colony (S.D. = 0.7). The mean size of colonies was 24.1 cm (S.D. = 17.5) 12 months after collection compared to more than double for the same colonies 12 months after pruning (58.4 cm (S.D. = 28.1)). Similarly, the colonies had 5.2 branches per colony (S.D. = 3.2) 12 months after initial collection compared to nearly double for the same colonies 12 months after pruning (9.5 branches per colony (S.D. = 3.5)). The yearly growth of colonies after collection was 21.9 cm yr^−1^ (S.D. = 15.6) compared to the yearly growth of the same colonies after pruning (55.6 cm yr^−1^ (S.D. = 27.0)). The annual productivity of colonies after initial collection was 4.2 cm (S.D. = 1.8) compared to the annual productivity of the same colonies after pruning (20.3 cm (S.D. = 8.8)). All of the growth metrics measured (i.e., growth, annual productivity, number of branches) were significantly higher for colonies after pruning (all genotypes grouped together, t test, p<0.05 for all metrics) (Fig. 4B). When the genotypes were examined individually, annual growth was significantly faster after pruning for 16 of 19 genotypes (Mann Whitney U test, p<0.05), while annual productivity was significantly greater after pruning for all 19 genotypes (Mann Whitney U test, p<0.05). The level of pruning (i.e., % of coral removed) was significantly and positively related to productivity (linear regression, p<0.05, R^2^ = 0.17).

## Discussion

*Acropora cervicornis* is an extremely fast-growing branching coral with annual productivity rates that often exceed 5 cm of new coral produced for every cm of existing coral in both Florida and the Dominican Republic. In addition to having prolific growth, this species benefits from high fragment survivorship coupled by the pruning vigor experienced by the parent colonies after fragmentation [16]. Here, we demonstrate that not only is the growth of donor colonies enhanced by pruning but that colonies can lose up to 95% of their tissue and skeleton through fragmentation and still have enhanced growth and recovery. The fast regrowth of damaged colonies after severe fragmentation has also been shown on wild populations of *Acropora palmata* after significant hurricane damage [24]. These life-history characteristics make acroporid corals highly successful nursery species and provide optimism for the potential role that active propagation can play in the their recovery.

Establishing clear relationships between coral colony size and growth has remained elusive due to the difficulties of measuring size and growth accurately, especially in natural conditions. The majority of the information published on this key relationship is based on studies that examine nubbins or colonies of a limited size range [25], or estimate colony growth based on: 1) extension rates measured from skeletal cores [26] or a subset of marked branches [27]; 2) 2-dimensional estimation of colony surface area based on field measurements or photographs [28], [29]; 3) 3-dimensional models [30], [31]; or 4) buoyant weights [32]. This is one of the first studies to report genotype-specific, whole-colony growth for a large number of colonies of a wide range of colony sizes, expanding on the patterns reported by Griffin et al. [10] based on six staghorn genotypes.

In this study, we document a significant positive linear relationship between colony size (TLE) and growth and branch development that is not surprising considering that staghorn corals grow through the creation of new branches and subsequent linear extension from apical ends [8]. Clark and Edwards [33] also showed a significant positive linear relationship between colony size and growth for three species of Pacific branching *Acropora* spp. However, this positive relationship becomes a decreasing exponential function when size and productivity are related for *A. cervicornis*. While more coral is being produced by bigger colonies, the rate at which new coral is produced normalized by the amount of existing coral declines as colonies grow, with maximum productivity rates recorded for small fragments and colonies. A similar pattern, but documented over a very narrow size range (1–4 cm TLE), was observed for *Acropora pulchra* by Soong and Chen [34], where extension rates increased with size but productivity of larger fragments was lower than that of smaller ones. Similarly, reductions in net growth with increasing colony size were documented by Hughes and Connell [35] and Yap et al. [36]. In corals, the resources allocated initially to branch extension may be shifted later to support radial growth (our observations indicate that colonies get thicker at the base as they grow), reproduction, and recovery from fragmentation, thus resulting in reduced productivity. An additional factor contributing to the reduction in productivity for this branching coral may be changes in branching patterns as colonies grow larger. If branching rates decrease over time due to physical limitation or within-colony crowding, linear growth rates would not be maintained. Within a coral propagation framework, results from the present study suggest that corals should be trimmed frequently to sustain productivity. While the optimal size to sustain high productivity will vary by species, the average annual productivity of staghorn colonies kept in nurseries in Florida and the DR dropped from> 5 cm for colonies <30 cm TLE to <3.2 cm for larger colonies. Lastly, considering the significant relationships demonstrated between colony size and growth and productivity in branching corals like *A. cervicornis*, caution should be exercised when comparing these metrics among treatments or even among different studies where initial fragment or colony size may be different.

The growth of *A. cervicornis* is influenced by the environmental setting where colonies are maintained. Coral nurseries are, as desired, locations of high productivity when compared to reef habitats where the productivity of transplanted, nursery-grown staghorn corals was, on average, 10% lower. Differences in productivity may be explained in part by considering that extensive resources are spent within nurseries to remove, manually, sediments deposited on colonies as well as coral predators and competitors like macroalgae and sponges [13]. Growth can also be influenced by propagation platform within the same habitat. Growing fragments on floating nursery structures that allow corals to develop branches in all directions was shown here to enhance branching and productivity and should be included by nursery managers as part of comprehensive propagation protocols. Indeed, some of the fastest growth rates reported for nursery-grown staghorn fragments are from floating nursery platforms in Puerto Rico where small fragments (4.4 cm TLE) grew an average of 52.5 cm in one year [10]. While the potential factors resulting in enhanced growth of corals suspended on ropes were not tested explicitly here, increased water motion has been shown to increase branching in *A. cervicornis* [37], [38] and growth in *Pocillopora damicornis* and *P. meandrina* [32].

The unique framework provided by our propagation program, based on the collection of coral genotypes from a wide range of “home” habitats and subsequent growth in nursery environments, allowed us to explore the interactive role of holobiont genotype and environment on staghorn coral growth [39]. Within common gardens (nurseries), corals exhibited a wide range of productivity values, and significant differences in growth between the fastest and slowest-growing genotypes. Genotypic-based variation in coral growth within the same environment has been documented previously for *Favia* and *Diploastrea* by Todd et al. [39], *Porites* by Forsman et al. [25], and *Stylophora* by Rinkevich [40] and Shaish et al. [41]. In contrast, no differences in growth were documented for distinct genotypes of *Madracis* by Bruno and Edmunds [42].

*Acropora cervicornis* is known to host a variety of algal symbionts in *Symbiodinium* clades A, C and D, with dominance varying depending on environment [21], [43], [44], [45]. The dominant symbiont in most shallow environments sampled to date appears to be A3 [43], [44], but as our data show, members of other clades can occasionally dominate in shallow water (see also [45]). Our study found C-dominated *A. cervicornis* to be uncommon (3 of 37 genotypes, 8%) in shallow reefs of Florida and the DR, but these symbionts are likely to be much more common in deep water populations [43], [44] which, to date, have been little studied. It is also possible that symbionts are more temporally and spatially variable than our results suggest. Additional within-colony sampling and long-term repeated sampling may reveal these associations to be more dynamic than our results suggest (but see [44]).

Corals containing different *Symbiodinium* can vary in their thermotolerance, especially corals dominated by certain *Symbiodinium* in clade D, including D1a in the Caribbean [46], [47], [48]. However, in our study, no colonies were found to be dominated by clade D *Symbiodinium* in Florida and no presence of D was found in colonies from the DR (assuming our limited sampling is representative of the symbiont communities found in each colony), and it is therefore unlikely that these symbionts would contribute significantly to the potential bleaching resistance of their hosts. However, corals with background, low abundance of various symbionts might recover more quickly from bleaching (i.e., be more resilient), especially if thermotolerant symbionts are present in their tissue. In this context, the presence of low-abundance clade D symbionts in Florida corals and the absence of D symbionts in DR corals might reflect differences in bleaching resilience, thermal regime, or disturbance (bleaching) history between these two populations. Monitoring the fate of symbionts during bleaching and recovery would help resolve the functional significance, if any, of background symbionts, which appear to be common in scleractinian corals [49]. C-dominated colonies might be expected to perform better under lower light conditions, compared with their A-dominated counterparts, but since these colonies co-existed at our sampling sites it is possible that, at the specific depths of our study, different *Symbiodinium* were equally beneficial to their coral hosts. Consequently, it may not be surprising to find that A- and C-dominated colonies have similar growth rates under these conditions, especially given our unbalanced statistical comparison (2 C-dominated genotypes vs. 22 A-dominated genotypes). A better test of physiological differences between these symbionts might be provided by transplanting colonies to deeper or shallower depths, or exposing them to combined heat and light stress to induce bleaching.

The realization that different coral genotypes can exhibit significantly different growth patterns within the same exact environment highlights the pressing need to incorporate, explicitly, genotype tracking into studies evaluating coral growth responses to environmental and experimental conditions. Without proper tracking, genotype influences on coral growth may mask or amplify a mean response if not taken into account in the sampling or experimental design. Moreover, with the possibility that colonies within patches or reefs can be clones produced by asexual propagation (prevalent in branching corals; [50]), the unbalanced inclusion of clone-mates in the estimation and comparison of growth patterns may raise issues related to lack of independence among samples and influence the statistical power to detect differences.

Our study is the first to document, in detail, the patterns of algal symbiont identity, growth, and productivity of asexually produced fragments of a large number of genotypes of the threatened Caribbean staghorn coral *Acropora cervicornis*. We show that even small fragments of this species are capable of extremely fast growth, but that productivity of developing colonies decline with colony size, indicating that regular pruning is required to maximize nursery productivity. In addition, large variability in growth was observed among coral genotypes maintained within a common environment, with an order-of-magnitude difference in growth between slow- and fast-growers. These findings have important implications for the propagation of corals and for the restoration of damaged reefs. While the identification of fast-growing coral genotypes may prompt practitioners to concentrate efforts on these “winners” to minimize time to ecosystem recovery, restoring genetic and genotypic diversity should also be considered as a key goal of restoration plans [51]. Moreover, further research is needed to fully understand the relationship between coral growth, algal symbiont identity, and resistance to stressors such as temperature anomalies and acidification. To date only a few coral genotypes have been tracked at both the nursery and outplanting phase of reef restoration and there still remains the possibility that coral growth by genotype is modulated by the environment and that “losers” within a nursery setting may become, over time, “winners” under different conditions experienced on reefs [39]. While documenting the reaction norm for corals remains a challenge, the expansion of *in situ* propagation programs [52] should be able to provide researchers with the ramets and genets required to conduct the challenging, large-scale transplantation experiments required to address this fundamental aspect of coral ecology and provide scientific support for the active recovery of this and other threatened species.

## Supporting Information

Figure S1 Field measurements and growth of staghorn fragment. Photographs of a staghorn fragment taken after deployment at the Florida nursery (left image) and after 6 months of growth (right image). The yellow segments show the measurements taken. The sum of all of the segments represents the Total Linear Extension (TLE) of the fragment. The fragment on the left has a TLE of 12 cm and 2 branches, the fragment on the right has a TLE of 32 cm and 8 branches. The annual growth of this fragment was calculated at 40 cm per year. The annual productivity was calculated at 3.3 cm of new coral/cm of initial coral. The images were taken by J. Herlan. The figure was composed based on the guidelines for the estimation of TLE proposed by Johnson et al. [13].
(TIF)

Data S1 Size and number of branches of the staghorn corals included in this study.
(XLS)
