# Supplementary figures and images for: Growth Dynamics of the Threatened Caribbean Staghorn Coral Acropora cervicornis: Influence of Host Genotype, Symbiont Identity, Colony Size, and Environmental Setting
Source: PLoS One. 2014 Sep 30;9(9):e107253. doi: 10.1371/journal.pone.0107253 (PMC4182308; doi:10.1371/journal.pone.0107253)

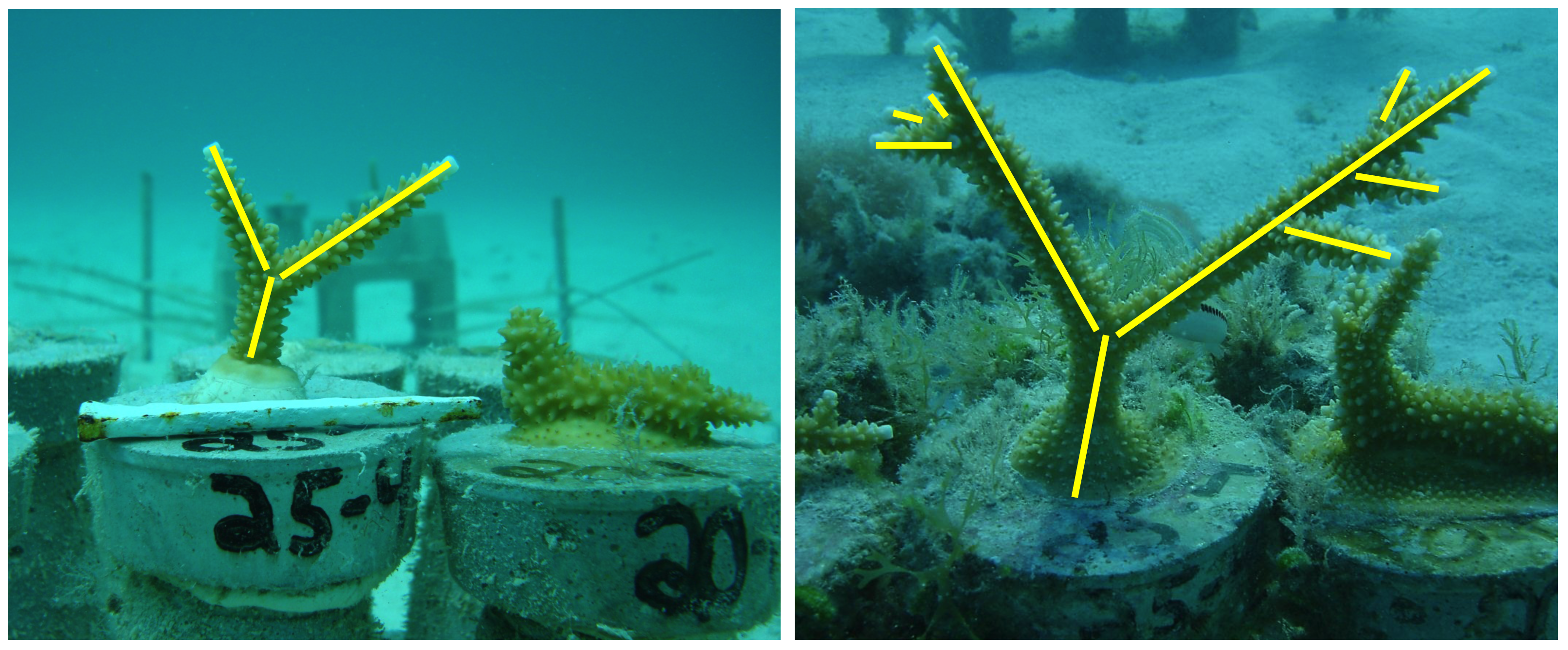

Supplement: Figure S1 — Field measurements and growth of staghorn fragment. Photographs of a staghorn fragment taken after deployment at the Florida nursery (left image) and after 6 months of growth (right image). The yellow segments show the measurements taken. The sum of all of the segments represents the Total Linear Extension (TLE) of the fragment. The fragment on the left has a TLE of 12 cm and 2 branches, the fragment on the right has a TLE of 32 cm and 8 branches. The annual growth of this fragment was calculated at 40 cm per year. The annual productivity was calculated at 3.3 cm of new coral/cm of initial coral. The images were taken by J. Herlan. The figure was composed based on the guidelines for the estimation of TLE proposed by Johnson et al. [13]. (TIF) [file pone.0107253.s001.tif]
